# Coastal proximity of populations in 22 Pacific Island Countries and Territories

**DOI:** 10.1371/journal.pone.0223249

**Published:** 2019-09-30

**Authors:** Neil L. Andrew, Phil Bright, Luis de la Rua, Shwu Jiau Teoh, Mathew Vickers

**Affiliations:** 1 Australian National Centre for Ocean Resources and Security, University of Wollongong, Wollongong, Australia; 2 The Pacific Community, Noumea, New Caledonia; 3 WorldFish, Penang, Malaysia; University of Vermont, UNITED STATES

## Abstract

The coastal zones of Small Island States are hotspots of human habitation and economic endeavour. In the Pacific region, as elsewhere, there are large gaps in understandings of the exposure and vulnerability of people in coastal zones. The 22 Pacific Countries and Territories (PICTs) are poorly represented in global analyses of vulnerability to seaward risks. We combine several data sources to estimate populations to zones 1, 5 and 10 km from the coastline in each of the PICTs. Regional patterns in the proximity of Pacific people to the coast are dominated by Papua New Guinea. Overall, ca. half the population of the Pacific resides within 10 km of the coast but this jumps to 97% when Papua New Guinea is excluded. A quarter of Pacific people live within 1 km of the coast, but without PNG this increases to slightly more than half. Excluding PNG, 90% of Pacific Islanders live within 5 km of the coast. All of the population in the coral atoll nations of Tokelau and Tuvalu live within a km of the ocean. Results using two global datasets, the SEDAC-CIESIN Gridded Population of the World v4 (GPWv4) and the Oak Ridge National Laboratory Landscan differed: Landscan under-dispersed population, overestimating numbers in urban centres and underestimating population in rural areas and GPWv4 over-dispersed the population. In addition to errors introduced by the allocation models of the two methods, errors were introduced as artefacts of allocating households to 1 km x 1 km grid cell data (30 arc–seconds) to polygons. The limited utility of LandScan and GPWv4 in advancing this analysis may be overcome with more spatially resolved census data and the inclusion of elevation above sea level as an important dimension of vulnerability.

## Introduction

The coastal zones of maritime countries are hotspots of human habitation and economic endeavor, with all the opportunities and risks that come with these activities [1,2]. This is particularly so in Small Island Developing States (SIDS) across the Indian, Atlantic and Pacific Oceans [3,4] where the ocean looms large as a source of food, wealth and cultural identity (e.g. [5,6]), but also as a source of acute and chronic threats. Tsunami, inundation and erosion caused by cyclone induced wave surge, and the long-term manifestations of sea level rise among other damaging phenomena all increase the exposure and vulnerability of coastal populations.

Understanding where people live in the dynamic coastal zone [7] is fundamental in a range of policy domains. Demand for such information may be divided into two broad inter-related categories. Firstly, such analyses provide the foundation of integrated analysis of risk and exposure to sea-based threats, including chronic risks associated with climate change (e.g. [2,8,9,10,11,12]), and acute risks from extreme weather events [2,13,14,15,16]. Distance from the coast is an important dimension of such analyses and, in the absence of elevation and other variables, is a proxy for vulnerability to sea-based threats. Another broad category of need is in the management of the coastal zone in face of multiple conflicting uses, including tourism, urban development and, increasingly, the alienation of small-scale fishing communities as more people migrate to the coastal zone [17,18,19,20,21].

Information required to map and analyze the spatial arrangement of people is usually derived from national population censuses and from global datasets. Although the spatial resolution of data is improving, historically, there has been a mismatch between demographic data collected in census enumeration areas and other relevant information, such as elevation, land cover, and vulnerability to natural hazards. SIDS present many of the challenges found in other developing countries in terms of infrequent and limited national survey data and a spatial resolution that is not fit for purpose to support policy development. As a result, most analyses in developing states rely to a large extent on publically available and often global data sources that provide data at relatively coarse resolution.

The Pacific region is home to 22 diverse Pacific Island Countries and Territories (PICTs), most of which share a dependence upon the ocean for food and economic development. Unless otherwise stated, the term ‘region’ refers only to the PICTs and not Pacific rim countries such as United States and New Zealand that are parties to regional organizations. Natural hazards, such as earthquakes, tsunamis and cyclones are ever present [14,15]. Four countries: Vanuatu, Tonga, Solomon Islands and Papua New Guinea (PNG), are among the world’s most disaster-prone nations. More chronic threats, from drought, saltwater intrusion of freshwater aquifers, and changing patterns in the productivity of fisheries from cyclic weather phenomena add to a challenging policy and planning landscape [12,16,22]. Four PICTs are low-lying coral atolls and reef islands: Kiribati, Tuvalu, Tokelau, and Marshall Islands, and are recognized as among the most vulnerable nations in the world to climate change. As Pacific societies respond and adapt to this complex opportunity and risk environment, there will be an increasing demand for information about where people live.

Gaps in information at appropriate scales are as evident in the Pacific SIDS as it is for other regions. National census and other demographic surveys are a large burden on national budgets, are infrequently done, and present major challenges for efficient and accurate data collection [23, 24]. Further, national surveys have traditionally allocated population homogeneously within enumeration areas, potentially biasing analyses through the so-called modifiable areal unit problem or MAUP [25]. The rapid development of geospatial methods and availability of satellite imagery and other sources of supplementary data improve the likelihood of minimizing the impact of MAUP and other limitations, including through the use of model-based dasymetric techniques [1,16,26,27].

In this paper we estimate the numbers and proportions of populations living close to the coast in the 22 PICTs, and to stratify these estimates by distance from the coast. We use a combination of national census data and two global datasets, the SEDAC-CIESIN GPWv4 [28,29] and the Oak Ridge National Laboratory LandScan [30,31]. National census data vary in detail among PICTs: the recently completed national census in Tonga gathered georeferenced household data, but the vast majority of PICTs have older, less reliable and precise information. Our analysis explores differences in population estimates using census data and global models, and develops a decision tree to guide future analyses based on the availability and method of national surveys.

## Methods

To meet existing demand from regional stakeholders and to illustrate our analysis we compare estimates of the numbers of people living within 1, 5, and 10 km from the coast. Most studies that estimate the size and disposition of coastal populations are framed within the concept of the low-elevation coastal zone (LECZ) [2,9,16], defined by elevations of generally 1–20 m above sea-level. Others use arbitrary categorizations that fit the context of the study (e.g. [32,33]). We have used the latter approach because (i) there is information available on elevation above sea level in only a few PICTS, and (ii) as in other regions, there is no universal definition of the ‘coastal zone’ in the region (see also, for example, https://coast.noaa.gov/czm/media/StateCZBoundaries.pdf). Further, our choice of zones and the scales of analysis were guided by the small sizes of many atolls and sampling units used to collect household data. As more geo-referenced information becomes available analyses will be refined and more sophisticated questions can be asked of the data, including, for example, relating exposure to seaward risk to household livelihood portfolios.

**Fig 1:**
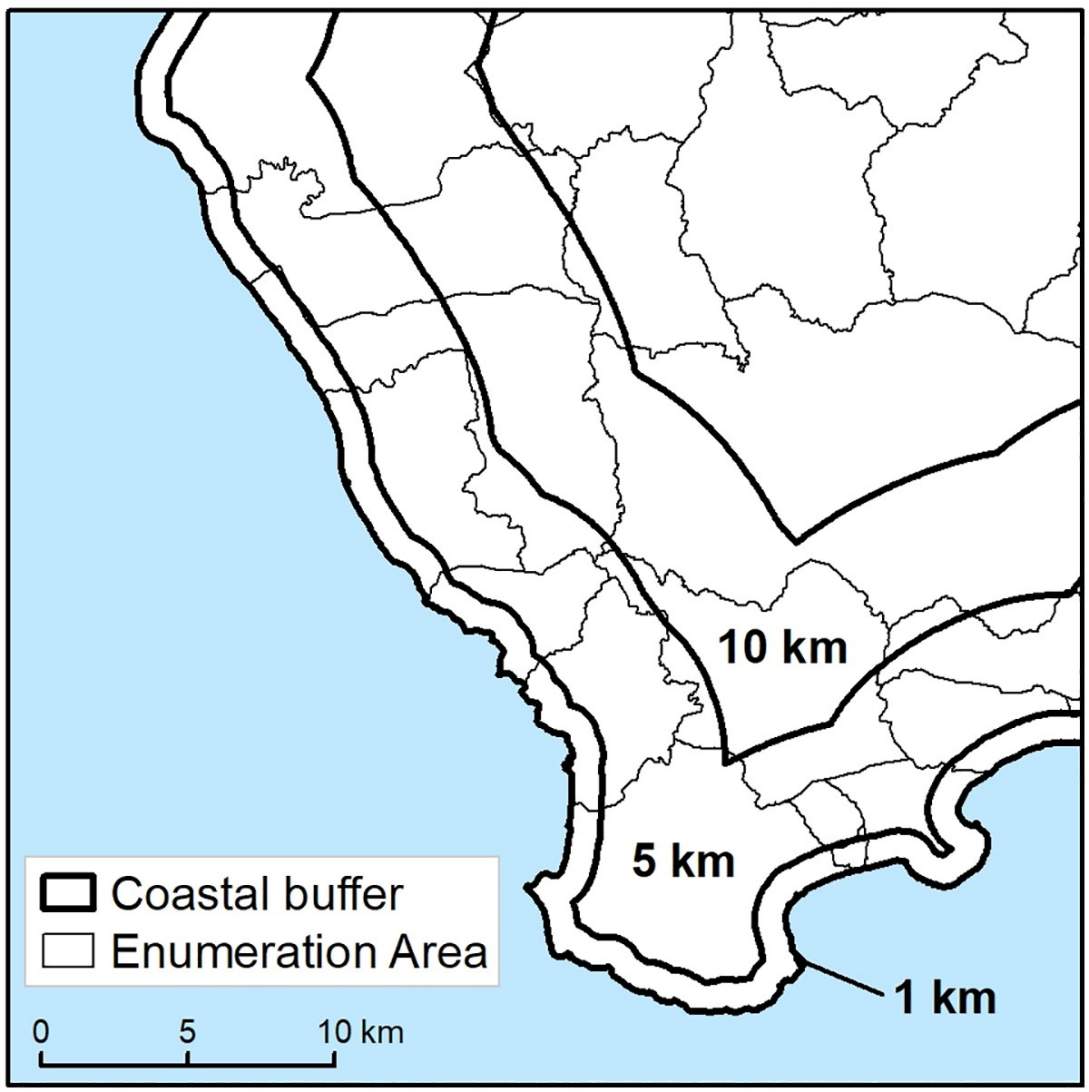
Zones used in analysis of coastal proximity of PICT populations. Zones of 1km, 5km, and 10km (black lines) overlaid onto local census enumeration areas in south-west Santo Island, Vanuatu. Enumeration areas that crossed boundaries were assigned a single zone based on the centroid of the enumeration area.

Three coastal zones at 1, 5, and 10 km were mapped onto every populated island for each PICT (Fig 1) using ArcGIS [34]. People residing within 1 km were considered to live on the coast, those within 5 km included those who could still easily walk to the coast and 10 km, those who interact with coastal communities (e.g. in terms of access to markets or other activities) and who would be able to easily get to the coast with some form of transport.

The two global datasets used to spatially distribute populations were the SEDAC-CIESIN Gridded Population of the World v4 (hereafter GPWv4; http://sedac.ciesin.columbia.edu/data/collection/gpw-v4) and the Oak Ridge National Laboratory LandScan^™^ (2015) (hereafter LandScan; http://web.ornl.gov/sci/landscan/index.shtml). GPWv4 [28,29] is a minimally modelled population dataset that uniformly distributes census data into 30 arc-second (approximately 1 km resolution at the equator) grid cells. Population data for censuses occurring between 2005 and 2014 were collected at the most detailed spatial resolutions available and then extrapolated to produce population estimates for 2015. LandScan [30,31] allocates population census data using dasymetric cartographic techniques where ancillary data such as land cover, roads, terrain slope, urban extent, and accessibility are used to model population density at 30 arc-second grid resolution within administrative boundaries. Both global datasets distributed populations within enumeration areas to 30 arc second cells (approximate 1 km^2^ at the Equator).

### Land and administrative boundaries

**Fig 2:**
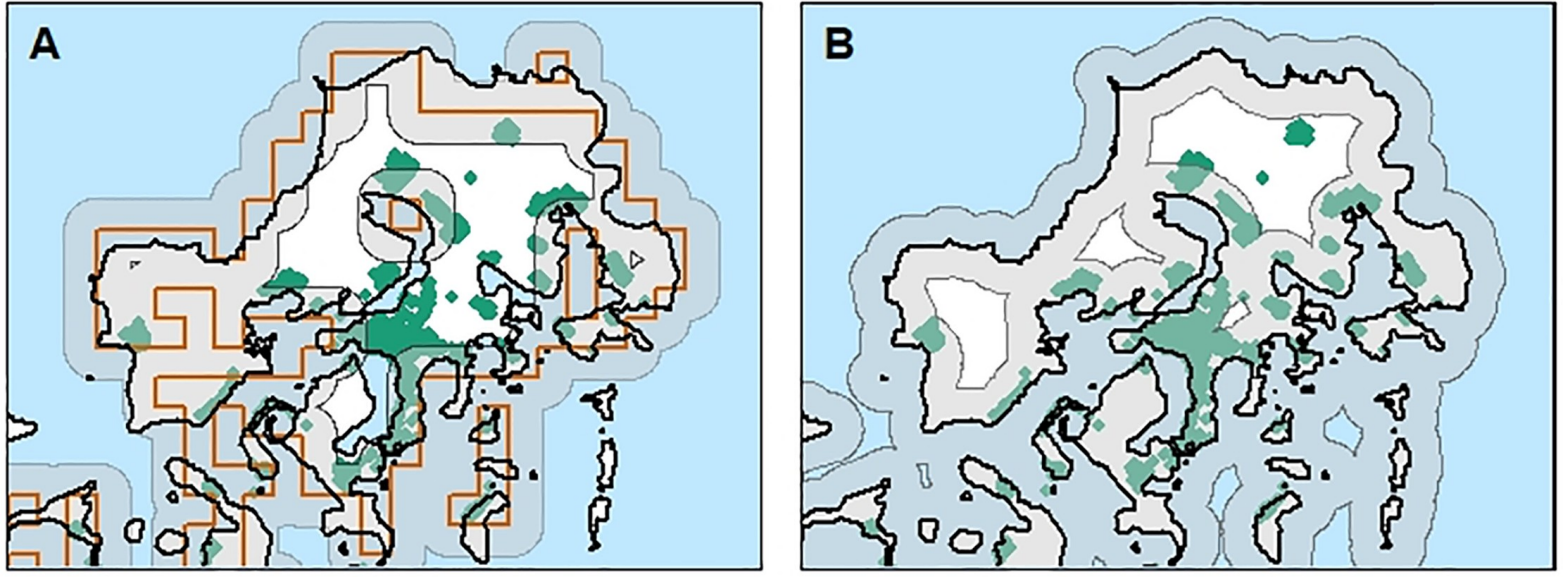
Differences in allocation of houses to 1 km zone using Landscan and GAUL boundaries illustrated using ArcGIS. (A) LandScan zone boundaries (brown) and associated 1 km zone miss-allocated many houses (green points) around inlet bays. (B) Allocations using GAUL land boundaries correctly allocated houses to zones.

Land administrative boundary and coastline data were extracted from the FAO Global Administrative Unit Layers (GAUL) network (www.fao.org/geonetwork/srv/en/-metadata.show?id=12691). For both global datasets, we overlaid the coastline boundaries of GAUL onto population data when defining the zones because LandScan geographic outlines were too coarse with respect to coastline boundaries for small islands (Fig 2). In particular, this meant that houses near inlet bays or on narrow isthmuses that misalign with the 30 arc-second grid would have been excluded by LandScan, but are included by coastal zones drawn using GAUL data (Fig 2). Enumeration areas that crossed zone boundaries were assigned a single zone based on the centroid of the enumeration area.

In each zone, we estimated the number of residents using the latest national census and from two global databases. Both global datasets had population estimates for 2015 and censuses were done between 2000 and 2015 (Table 1). Each nation was in control of its own census protocol, though often guided by the Statistics for Development Division of the Pacific Community, and the two global databases were curated by independent agencies. In general, national population data could not be projected to 2015 at subnational level, as we lacked spatial resolution for vital statistics and within-country migration patterns. We therefore normalised population estimates to the total coastal population of each dataset.

**Table 1:** Summary of coastlines, populations and data sources for PICTs. Population estimates from https://sdd.spc.int/topic/population. Coastline lengths from the World Vector Shoreline database at 1:250.000 scale and The World Factbook (https://www.cia.gov/library/publications/resources/the-world-factbook/index.html). Census unit abbreviations: EA = Enumeration Area and LLGA = Local Level Government Area. Analysis methods vary depend on the data availability (see text for further details).

Country	ISO3	Land area (km^2^)	Coastline (km)		Population	Census Year	Census unit	Source	Methods
**MELANESIA**	** **	**540,260**	**41,471**	** **	**7,046,717**	** **	** **	** **	** **
Fiji	FJI	18,333	4,638	a	837,271	2007	EA	Fiji NSO	2
New Caledonia	NCL	18,576	3,624	a	268,767	2014	EA	ISEE	3
Papua New Guinea	PNG	462,840	20,197	a	5,190,786	2000	LLGA	PNG NSO	3
Solomon Islands	SLB	28,230	9,880	a	515,870	2009	EA	Sol NSO	2
Vanuatu	VUT	12,281	3,132	a	234,023	2009	EA	Vanuatu NSO	2
**MICRONESIA**	** **	**3,156**	**8,519**	** **	**506,541**	** **	** **	** **	** **
FSM	FSM	701	1,295	a	102,843	2010	EA	FSM NSO	2
Guam	GUM	541	126	b	159,358	2010	EA	US Census Bureau	3
Kiribati	KIR	811	1,961	a	109,693	2015	EA	Kiribati NSO	100% coastal
Marshall Islands	MHL	181	2,106	a	53,158	2011	EA	RMI NSO	100% coastal
Nauru	NRU	21	30	b	9,945	2011	EA	Nauru NSO	3*
Northern Mariana Islands	MNP	457	1,482	b	53,883	2010	Village	CNMI NSO	2
Palau	PLW	444	1,519	b	17,661	2015	EA	Palau NSO	2
**POLYNESIA**	** **	**8,126**	**7,807**	** **	**652,976**	** **	** **	** **	** **
American Samoa	ASM	199	116	b	55,519	2010	County	US Census Bureau	3
Cook Islands	COK	237	120	b	14,974	2011	EA	Cooks NSO	3
French Polynesia	PYF	3,521	5,830	a	268,207	2012	Commune	ISPF	1
Niue	NIU	259	64	b	1,460	2011	EA	Niue NSO	3
Pitcairn Islands	PCN	47	51	b	57	2012			100% coastal
Samoa	WSM	2,934	463	a	187,820	2011	EA	Samoa NSO	2
Tokelau	TKL	12	101	b	1,411	2011	EA	Tokelau NSO	100% coastal
Tonga	TON	749	909	a	100,691	2011	Block	Tonga NSO	1
Tuvalu	TUV	26	24	b	10,640	2012	EA	Tuvalu NSO	100% coastal
Wallis and Futuna	WLF	142	129	b	12,197	2013	Village	WLF NSO	2

### Allocation of census data

Population data collected from national censuses were collected using different methods and so varied in spatial precision. Countries were analysed with one of four methods depending on the availability of data:

- **Method 0.** In five of the smallest PICTs the land area of all populated islands fell within the 1 km zone. No analyses or assumptions were required. This method was used in the following PICTs: Kiribati, Marshall Islands, Pitcairn Islands, Tokelau, and Tuvalu. All except Pitcairn Islands are comprised solely of coral atolls and coral reef islands (defined as low-lying accumulations of unconsolidated, or poorly lithified, carbonate sands and gravel deposited on coral reef platforms [35].
- **Method 1**. Households were GPS-located and each household had specific census data, allowing the population in each coastal zone to be counted directly and hence this is the ‘gold standard’ for mapping population distribution. Data were geo-rectified using local coordinate reference systems. This method assumed that both GPS and census data were accurate and precise. PICTs: Tonga and French Polynesia.
- **Method 2**. Households were GPS-located. Population data was only available down to enumeration area, extracted from census datasets. The population data and number of households were used to calculate an average mean household size in each enumeration area, which was allocated to each GPS-located house. This method resulted in all households in a given enumeration area approximating the same household size. Coastal zone populations were calculated by selecting all households falling within each of them Data were geo-rectified using local coordinate reference systems. PICTS: Federated States of Micronesia (FSM), Fiji, Commonwealth of the Northern Mariana Islands (CNMI), Palau, Samoa, Solomon Islands, Vanuatu, and Wallis and Futuna.
- **Method 3**. Population data were available for each enumeration area with no GPS household location data available. The population was averaged by unit area across a given enumeration area. Vector analysis allowed us to precisely determine the proportion of each enumeration area, and hence each enumeration area’s population, within each zone. Data were georectified using the ESRI World Cylindrical Equal Area (WKID: 54034) projection to preserve area. This method assumed the population was uniformly distributed within the enumeration area. PICTs: American Samoa, Cook Islands, Guam, New Caledonia, Niue, Nauru, and PNG. Nauru was unique as only three enumeration areas fell outside the 1 km zone, and these were manually allocated to the 5 km Coastal zone.

Population estimates in the grid cells from the LandScan and GPWv4 data sets that bridged zone boundaries were allocated to a Coastal zone based on the location of the centre of the cell. All data are presented as raster images on a 30 arc-second grid which is the native resolution of the LandScan and GPWv4 datasets. The logic tree used to generate population estimates from methods 1–3 is summarized in S1 Fig.

More detail about how the coastal buffers were created and population figures calculated can be found at the protocols.io repository (http://dx.doi.org/10.17504/protocols.io.59yg97w).

### Effect of differences in census date

We conducted an analysis, using Fiji, to assess the impact of such differences in the year of census. We used data from 2007 to project a population for 2015 using birth/death rate data at the scale of the *tikina*, the local meso-level administrative unit. We calculated mean household population for each *tikina*, and allocated this value to household GPS data from the 2007 census. We then used these data to measure the number of people in each zone as if they were any other Method 2 data. We also had projected 2015 populations for both of the global databases to compare 2007 census, 2015 projected, and the two (LandScan and GPWv4) 2015 global-projected estimates. This comparison allowed us to quantify the effect of using census data from a range of dates. Secondly, by overlaying the global datasets with our projected 2015 population, we were also able to visualize where global projections over- or under-estimated population density.

## Results

### Proportions of populations living near the coast

**Fig 3:**
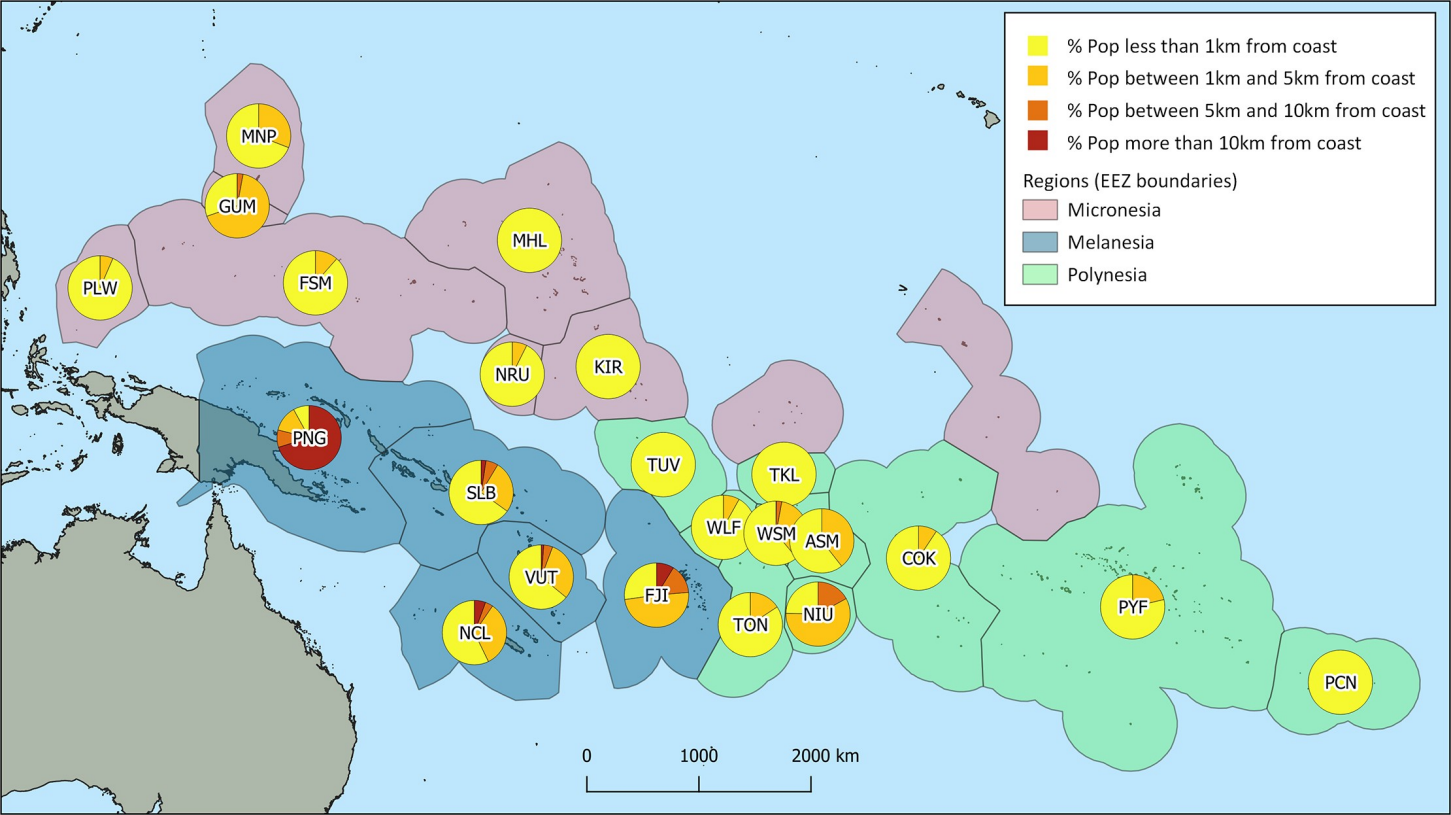
Proportions of households within 1, 5 and 10 km from the coast in 22 PICTSs. Proportions of households in each of the three buffers (see legend). See Table 1 for interpretation of the three digit ISO country codes.

Unsurprisingly, regional patterns in the proximity of Pacific people to the coast are dominated by PNG. Taken as a whole, about half of Pacific people living in the PICTs reside within 10 km of the coast but this jumps to 97% when PNG is excluded (Fig 3, Table 2, S1 Table). Closer to the coast, patterns reflect these same differences–slightly more than a quarter of Pacific people live within a km of the coast, but without PNG this increases to slightly more than half (Table 2, S1 Table). Excluding PNG, 90% of Pacific Islanders live within 5 km of the coast.

**Table 2:** Percentage of populations within 1, 5 and 10 km zones in PICTS, sub-regions and region. Estimates are derived from census, GPWv4 and Landscan. Where estimates differed by less than 5% only the census estimate is provided, otherwise the census estimate is bracketed as ‘census (GPWv4, Landscan)’. A complete listing of estimates is provided in S1 Table.–indicates that all of the land area is within the boundary of the next smaller zone. * Population as per most recent census (see Table 1).

		% in zone		
Country and sub-region	Population*	1km	5km	10km
** MELANESIA**	**7,046,717**	**18 (12, 27)**	**38 (30, 41)**	**47 (39, 47)**
**MELANESIA (excluding PNG)**	**1,855,931**	**47 (38, 56)**	**85**	**94**
Fiji	837,271	27 (32, 41)	76 (82, 80)	91
New Caledonia	268,767	57 (43, 57)	90 (66, 86)	94 (81, 96)
Papua New Guinea	5,190,786	8 (5, 17)	21 (16, 28)	30 (25, 33)
Solomon Islands	515,870	65 (42, 74)	91 (84, 92)	98
Vanuatu	234,023	64 (46, 68)	94	99
** MICRONESIA**	**506,541**	**72**	**99**	**100**
FSM	102,843	89 (85, 94)	100	100
Guam	159,358	30 (27, 35)	97	100
Kiribati	109,693	100	-	-
Marshall Islands	53,158	100	-	-
Nauru	9,945	93 (70, 93)	100	-
CNMI	53,883	69 (54, 77)	100	100
Palau	17,661	93 (81, 87)	100	100
** POLYNESIA**	**652,976**	**74 (39, 78)**	**99 (80, 99)**	**100**
American Samoa	55,519	61 (64, 72)	100	-
Cook Islands	14,974	91 (57, 91)	100	100
French Polynesia	268,207	79 (36, 83)	100 (77, 100)	100
Niue	1,460	25 (27, 63)	83 (86, 100)	100
Pitcairn Islands	57	100	-	-
Samoa	187,820	61 (17, 69)	97 (86, 97)	100
Tokelau	1,411	100	-	-
Tonga	100,691	84 (58, 77)	100	-
Tuvalu	10,640	100	-	-
Wallis and Futuna	12,197	92 (60, 88)	100	-
** PACIFIC region**	8,206,234	26 (16, 33)	47 (36, 48)	54 (45, 54)
** PACIFIC (excluding PNG)**	3,015,448	57 (43, 64)	90 (84, 91)	97

In Micronesia and Polynesia only two populated islands have land more than 10 km inland from the ocean, and only six have land more than 5 km from the coast. Including the landmass of Papua New Guinea, all populated Melanesian islands have land more than 10 km inland from the coast.

In Polynesia, the largest islands, Savai’i and Upolu (Samoa), Tahiti (French Polynesia), and Niue have interiors that are further than 5 km from the coast, but these areas are mostly rugged and uninhabited (Table 2). As a result, all of the sub-region’s population lives within 5 km of the coast and nearly 75% live within a kilometer of the ocean (Fig 3). All of the population in the coral atoll nations of Tokelau and Tuvalu live within a kilometer of the ocean. All of Pitcairn’s 57 residents live on the small island of Pitcairn—the much larger Henderson Island is unpopulated.

Micronesia has a greater proportion of coral atolls than Polynesia and Melanesia and almost everyone lives within 5 km of the ocean. The largest island in the subregion, Pohnpei (Federated States of Micronesia) has a rugged and uninhabited interior and the census allocated no population there. A small area on the island of Guam is more than 5 km from the coast, but only 3% of the population lives there.

In Melanesia, 47% live within 10 km of the coast, and when PNG was excluded, this rose to 94%. The many large volcanic islands of the sub-region support a large proportion of the Pacific region’s population, along with barrier reef islands and coral atolls. Including PNG, 38% of the population live within 5 km of the coast; this rose to 85% excluding PNG. In Solomon Islands and Vanuatu, over 60% lived within 1 km, though this was much less in New Caledonia (57%), Fiji (27%), and PNG (8%).

### Comparison of disaggregation methods

**Fig 4:**
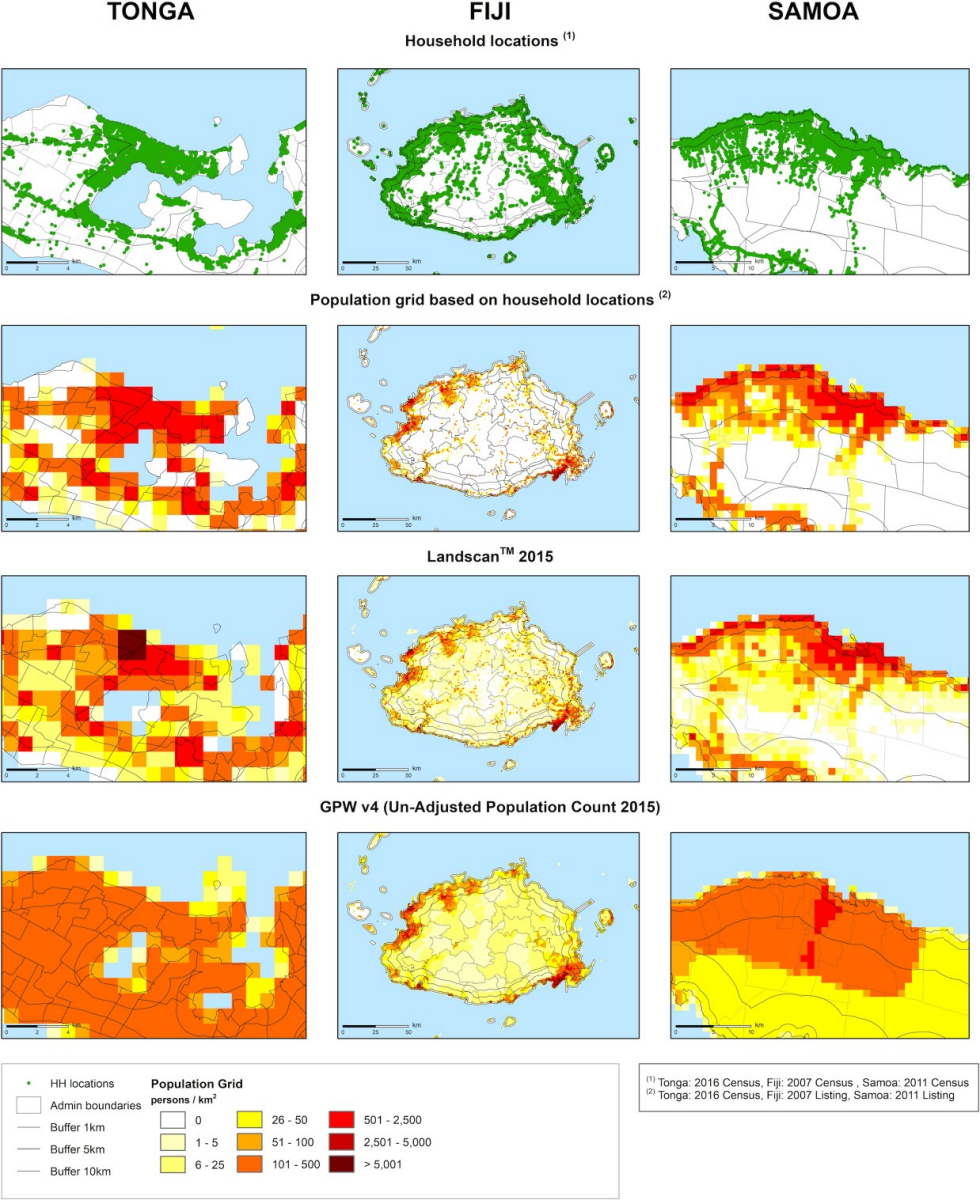
Comparison of methods for disaggregating populations to enumeration areas and zones. Known household locations shown in the top row (green) translated to high-precision estimates of population (second row), when population was known. LandScan estimates of population distribution overestimated populations in urban centres (row 3). GPWv4 did not favour urban populations, instead, the population was averaged across areas (row 4). The maps show the islands of Tongatapu (Tonga), Viti Levu (Fiji), and part of Upolu (Samoa).

Differences in allocation of households and people to zones using Method 1 and 2, and estimates derived from LandScan or GPWv4 were consistent with the nature of the algorithms used to allocate population within grids (Fig 4). Directly mapping household location and census data yielded the most accurate population distributions, while the Landscan and GPWv4 models had contrasting biases: Landscan tended to model an under-dispersed population, overestimating numbers in urban centers and underestimating population in rural areas (Fig 4, third row). In contrast, GPWv4 tended to over-disperse the population, resulting in a smoothed population distribution (Fig 4, bottom row).

**Fig 5:**
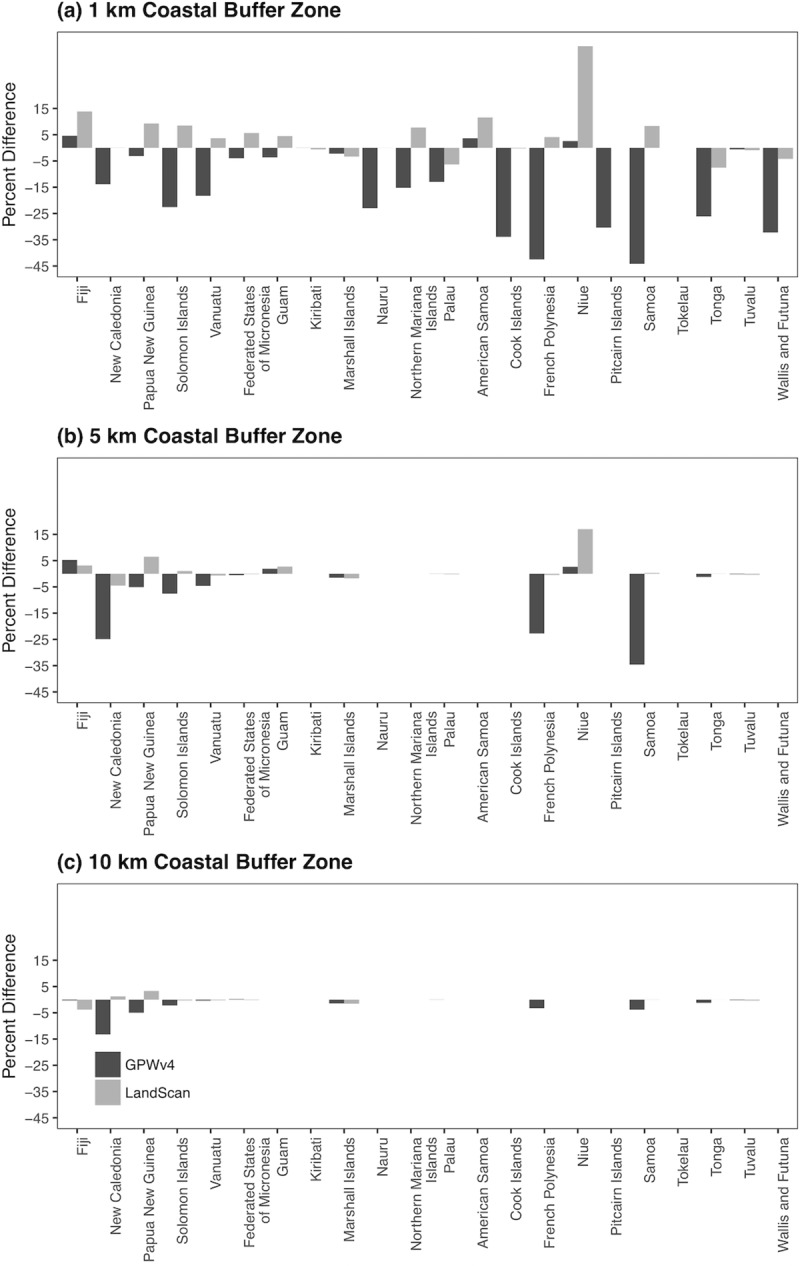
Differences in allocation between global population datasets. Percent differences between estimates of the population in (a) 1 km, (b) 5 km, and (c) 10 km buffer zone estimated by GPWv4 and LandScan and estimates using the census method.

The effect of the different methods were most apparent in the 1 km Coastal zone in all PICTS (Fig 5A), as well as highly apparent in the 5 km Coastal zone in the Melanesian PICTs (Fig 5B). When comparing the global databases with national census data (S1 Table) we found that, for the 1km zone, LandScan overestimated the proportion of the population by more than 5% for 8 out of 22 countries (Fig 5).

As the area of zones increased, LandScan and GPWv4 proportions converged towards National Census data estimates. In addition, different estimates of the proportion of people living within 10 km of the coast converged (Fig 5C, S1 Table). In general, LandScan over-estimated the percentage of population in the smallest zone by more than 5% for 8 out of 22 PICTs (Fig 5). The difference was greatest in Niue (38%), and Fiji (14%). In contrast, the GPWv4-derived estimates underestimated the proportion of the population living within 1 km of the coast in 12 out of 22 PICTs (Fig 5A). Deviations between GPWv4 and national census estimates were greatest in Polynesia: Samoa (44%), French Polynesia (42%), Cook Islands (34%) and Pitcairn Islands (30%). In Micronesia, Nauru was the highest with 23% deviation.

### Comparison of census methods

Only for Tonga were we able to compare the results from Method 1 and Method 2 data. In this case, the models agreed very closely. For other countries, we were able to compare Method 2 and Method 3. In general, models of Method 3 data underestimated populations in the 1km and 5km zones (Fiji, Solomon Islands, Vanuatu, Palau, and Samoa). For three PICTs, this was untrue: Federated States of Micronesia, Northern Mariana Islands (where Method 2 was very similar to Method 3), and Wallis and Futuna (Method 3 > Method 2). Generally, GPWv4 underestimated the proportion of people living in the 1 km and 5 km zones, though this was not true for Fiji or Niue. By contrast, the LandScan model tended to slightly overestimate proportions of populations in those zones.

## Discussion

UNEP [36] categorizes people as ‘coastal’ if they live less than 100 km from the sea. By that measure, almost all PICT residents, including in PNG, are coastal. Further, excluding PNG, almost all residents of PICTs live within 10 km of the coast and in five coral atoll nations (Kiribati, Marshall Islands, Tokelau, and Tuvalu) everyone lives with a km of the sea, as do nearly three quarters of Micronesians. Partially driven by the size and geology of their islands, a greater proportion of Melanesians live more than 10 km from the sea; PNG was the clear outlier with over 70% of the population living more than 10 km from the coast.

Although we have advanced understanding of the vulnerability to ocean-derived threats in the 22 PICTs, further resolution and greater policy relevance is constrained by data availability, both in terms of greater spatial resolution of households and their elevation above sea level as an important dimension of exposure. Clearly, national population census data georeferenced at the household level (including number of occupants), combined with ancillary data on elevation above sea level provides the best platform for future analyses. Recent Population censuses in Tonga and Vanuatu completed in 2016 have georeferenced households, as will the Kiribati household and income and expenditure survey in 2019.

In the meantime, for most countries in the region and possibly for other developing countries, analyses will rely on stitching together data of varying resolution and quality, including Landscan and GPWv4, among other sources. LandScan and GPWv4 data provide a significant source of information about where people live. Both sources are, however, publically available only at 30 arc-second scale and have inaccuracies that are predictable consequences of the models used. Unsurprisingly, differences among data sources diminished and converged on estimates from the census as greater proportions of the population were captured in the 1, 5 and 10 km zones. Most islands in the PICTs are less than 20 km across so most residents cannot be farther than 10 km from the coast.

Global data sets such as LandScan and GPWv4 provide invaluable sources of information, particularly in the developing world where there are limited alternatives. Nevertheless, at the scale of analysis needed to advance this work within the particular geographies of small Pacific islands they will be of limited utility. The LandScan model concentrated population into urban areas and where infrastructure (roads, night-time lights and so forth) was most developed—in all instances this coincided with development in the coastal zone. This model was not well suited to rural coastal villages, many of which do not have roads or electricity. Conversely, census method 3 and GPWv4 averaged populations across the grid cells and so over-dispersed population in rural areas. Errors from both GPWv4 and LandScan were greatest in the 1 km zone where finer scale accuracy was most needed. Other global datasets, such as WorldPop [27,28] have finer grids but were not available for PICTs at the time of writing.

In addition to allocation errors introduced by the global models, errors were introduced as an artefact of allocating 1 km x 1 km grid cell data (30 arc–seconds) to polygonal zones. The population in each grid cell was estimated, and each grid cell was then classified as being within the 1, 5, or 10 km zone based on the location of the center of the grid cell. Grid cells along the border of a zone therefore have a 50% chance of being classified as either in or out. The method was consistent among the three zones, but a by-product of the geography of islands is that the border for the 1 km zone is longer than the border for the 5 km, which is longer than that for 10 km. This means there were more classification errors for the 1 km zone than the 5 km zone than the 10 km zone. Simultaneously, the area for the 1 km zone in most PICTs was smallest and, therefore the impact of this error greatest. These effects are a predictable consequence of the weighting method used to allocate population to grids within enumeration areas [28].

Elevation is often incorporated in analyses of exposure and vulnerability using the concept of the Low Elevation Coastal Zone (LECZ), which is defined as ‘land area contiguous with the coastline and less than 10 m elevation’ [2,9,16]. Obtaining elevation data at the household scale and the disposition of rural and urban people are next steps in this analysis. As more people move toward the coast and urbanize, inclusion of LECZ data in national disaster vulnerability analyses will significantly improve predictions and disaster risk scenarios.

Pacific Island countries and territories are poorly represented in global analyses of exposure and vulnerability to seaward risks. The 22 sovereign nations and territories are usually lumped with SIDS globally, or lumped with East Asia or even Asia, are mis-categorized, or excluded from global analyses and summaries altogether because data are unavailable or they are too small (e.g. [2]). Neumann et al. [2] concluded that 55% of PICT populations in 2000 (excluding PNG– 8% with PNG) were found in the LECZ. Based on our estimate that 95% people live within 5 km of the coast and on knowledge of the geography of the region, we find the Neumann et al. (op. cit.) regional and national estimates improbably low and hypothesize that the great majority of people living in the Pacific region reside in the LECZ of those PICTs. We know that islands in the four coral atoll countries (Kiribati, Tuvalu, Marshall Islands and Tokelau) and many populated atolls and reef islands in other PICTs (e.g. Carteret atoll in PNG, Ontong Java in Solomon Islands, and Ouvea in New Caledonia) are wholly within the LECZ. The mountainous interiors of many populated islands (e.g. Moorea and Papeete in French Polynesia) and presence of active volcanoes on others (e.g. Ambae and Tanna in Vanuatu) force people to live on the coastal fringe. Cultural preferences and identity, urbanization and economic opportunity, among other drivers also pull people to the coastal fringes to live and work [18,37].

PICTs are inconsequential in the arithmetic of global summaries of exposure and vulnerability to seaward hazards [2,16]. More coastal people may be found in a single district in the LECZ of southwest Bangladesh than in the whole Pacific region. Nevertheless, the exclusion of these 22 countries and territories further marginalizes them from global narratives, particularly concerning the consequences of climate change. The results presented here and data layers available online at http://sdd.spc.int/mapping-coastal will hopefully promote better inclusion of Pacific countries and territories in global summaries, lists of vulnerable countries, and improve national SDG reporting. Were that to happen, PICTS will feature prominently in ‘top ten’ lists of exposed and vulnerable nations and territories.

## Supporting information

S1 Fig Logic tree. Comparison of processing for different methods (depending on how data were collected) and global population grids.
(TIF)

S1 Table Population figures for inhabitants within the three coastal zones (1 km, 5 km, and 10 km) across the PICTs. Data from national census, Oak Ridge National Laboratory LandScan and SEDAC-CIESIN GPWv4.
(XLSX)
