# Supplementary figures and images for: Coastal proximity of populations in 22 Pacific Island Countries and Territories
Source: PLoS One. 2019 Sep 30;14(9):e0223249. doi: 10.1371/journal.pone.0223249 (PMC6768456; doi:10.1371/journal.pone.0223249)

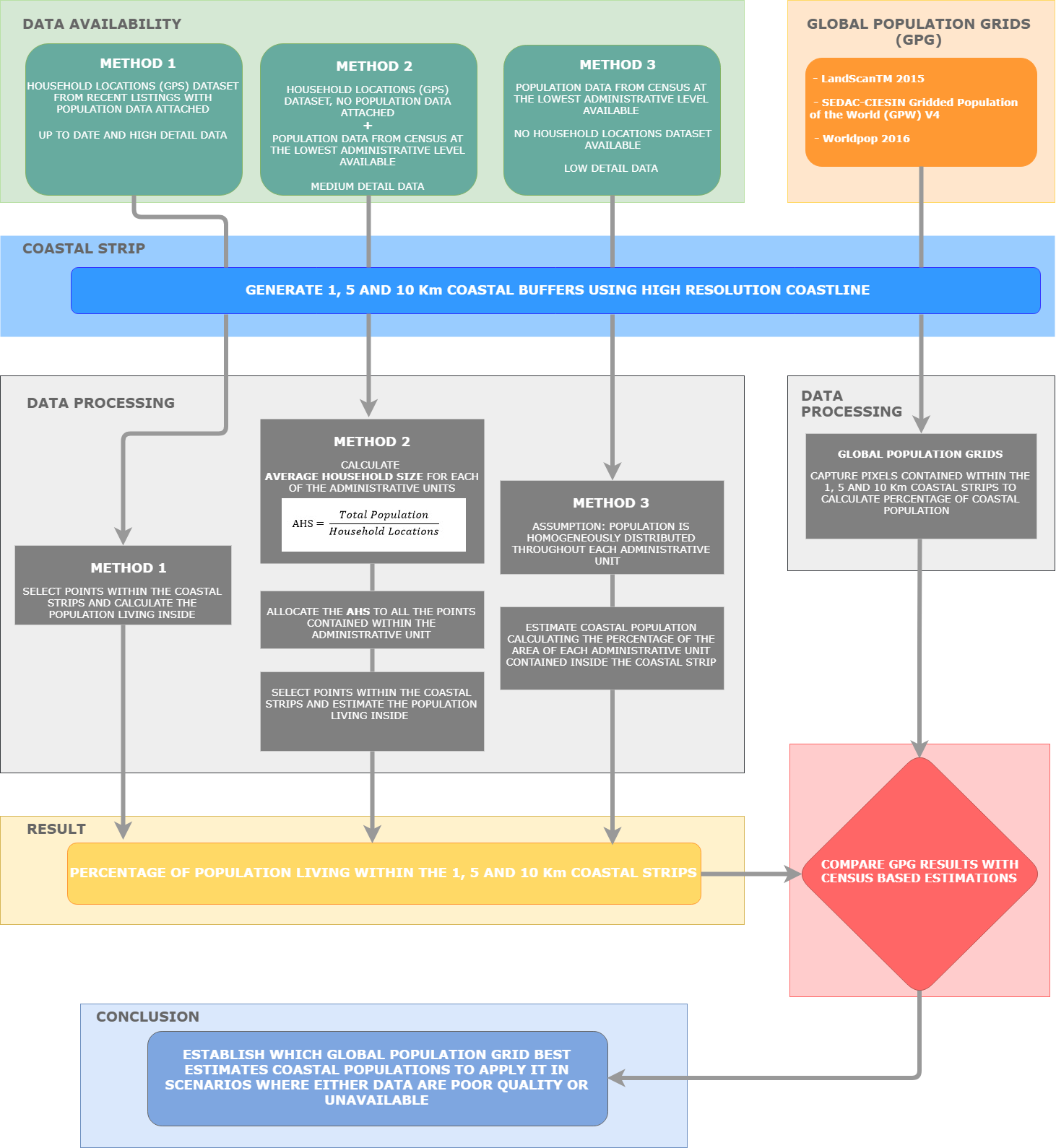

Supplement: S1 Fig — Comparison of processing for different methods (depending on how data were collected) and global population grids. (TIF) [file pone.0223249.s001.tif]
